# Feasibility, effectiveness, and safety of endoscopic vacuum therapy for intrathoracic anastomotic leakage following transthoracic esophageal resection

**DOI:** 10.1186/s12876-021-01651-6

**Published:** 2021-02-16

**Authors:** Chengcheng Christine Zhang, Lukas Liesenfeld, Rosa Klotz, Ronald Koschny, Christian Rupp, Thomas Schmidt, Markus K. Diener, Beat P. Müller-Stich, Thilo Hackert, Peter Sauer, Markus W. Büchler, Anja Schaible

**Affiliations:** 1 Department of Gastroenterology, Heidelberg University Hospital, University of Heidelberg, Im Neuenheimer Feld 410, 69120 Heidelberg, Germany; 2 Department of General, Visceral and Transplantation Surgery, Heidelberg University Hospital, Heidelberg, Germany

**Keywords:** Anastomotic leakage, Endoscopy, Endoscopic vacuum therapy, Negative-pressure therapy, Postoperative complications

## Abstract

**Background:**

Anastomotic leakage (AL) in the upper gastrointestinal (GI) tract is associated with high morbidity and mortality rates. Especially intrathoracic anastomotic leakage leads to life-threatening complications. Endoscopic vacuum therapy (EVT) for anastomotic leakage after transthoracic esophageal resection represents a novel concept. However, sound clinical data are still scarce. This retrospective, single-center study aimed to evaluate the feasibility, effectiveness, and safety of EVT for intrathoracic anastomotic leakage following abdomino-thoracic esophageal resection.

**Methods:**

From March 2014 to September 2019 259 consecutive patients underwent elective transthoracic esophageal resection. 72 patients (27.8%) suffered from AL. The overall collective in-hospital mortality rate was 3.9% (n = 10). Data from those who underwent treatment with EVT were included.

**Results:**

Fifty-five patients were treated with EVT. Successful closure was achieved in 89.1% (n = 49) by EVT only. The EVT-associated complication rate was 5.4% (n = 3): bleeding occurred in one patient, while minor sedation-related complications were observed in two patients. The median number of EVT procedures per patient was 3. The procedures were performed at intervals of 3–5 days, with a 14-day median duration of therapy. The mortality rate of patients with AL was 7.2% (n = 4). Despite successfully terminated EVT, three patients died because of multiple organ failure, acute respiratory distress syndrome, and urosepsis (5.4%). One patient (1.8%) died during EVT due to cardiac arrest.

**Conclusions:**

EVT is a safe and effective approach for intrathoracic anastomotic leakages following abdomino-thoracic esophageal resections. It offers a high leakage-closure rate and the potential to lower leakage-related mortalities.

*Trial registration:* This trial was registered and approved by the Institutional Ethics Committee of the University of Heidelberg on 16.04.2014 (Registration Number: S-635/2013).

## Introduction

Anastomotic leakage is a severe and life-threatening complication that occurs after esophageal resections. Especially intrathoracic leakages often lead to mediastinitis, pneumonia, bronchoesophageal fistulas, and sepsis [1–7]. The reported incidence of intrathoracic anastomotic leakages after esophageal resections vary widely from 1 to 35% [8–14]; reported mortality rates range from 7.2 to 60% [15–19]. After an esophageal resection, the presence of an anastomotic leakage doubles the mortality rate [15]. Several multicenter studies have identified anastomotic leakage as a strong independent prognostic factor for long-term survival [20].

The treatment of anastomotic leakages remains to be an interdisciplinary challenge. A delay in therapy for more than 24 h is associated with a threefold increase in mortality rates [21, 22]. To date, several treatment strategies for anastomotic leakage after esophageal surgeries are available. The therapeutic standards differ widely between centers and geographic regions. In cases without mediastinitis and those with only small leakages, a conservative approach may be undertaken. This involves treating the patient with antibiotics, maintaining *nil per os,* giving parenteral nutrition temporarily, or applying endoscopic metal clips or over-the-scope clips (OTSC) for defect closure [23–26]. For cases with larger leakages, the placement of self-expanding covered metal or plastic stents (SEMS or SEPS) has served as the first-line therapy [26–29]. The reported success rates of stent therapy range between 65 and 91% [26, 30, 31]. However, common complications have been observed. These include stent migration with a consequent inadequate defect closure, bleeding, local necrosis of the esophagus by stent pressure, ingrowth of the stent making later stent removal impossible, and development of an aortoesophageal fistula [30, 31]. The reported mortality rates of stent therapy vary widely and range from 0 to 83% [13, 32–35]. In case of an unsuccessful stent therapy and the development of septic conditions, esophageal diversion and cervical esophagostomy serve as *ultima ratio* [12, 23, 36].

Recently, endoscopic vacuum therapy (EVT) was established as the initial therapy for anastomotic leakages after esophageal resections. With regards to the upper GI tract, Wedemeyer et al. described, for the first time in 2008, the endoscopic insertion of a polyurethane sponge to the defect side and the application of an external vacuum [37]. The negative pressure therapy decreased and prevented bacterial contamination of the wound and promoted perfusion and granulation of the defect. Hence, the combination of the EVT with defect closure and an effective internal drainage were introduced. Different case reports or case series show favorable results to EVT in terms of sealing rates. Reported treatment success ranges from 66.7 to 100% [38, 39]. However, evidence on EVT application in the upper GI tract is still rare and mostly based on small retrospective study cohorts with heterogeneous esophageal defects of different etiologies, e.g. benign perforations and postoperative anastomotic leakages [12, 24, 25, 39].

To date, there is still no consensus on the therapeutic regimen of intrathoracic anastomotic leakages. The aim of this study was to evaluate the feasibility, effectiveness, and safety of EVT in terms of success rate and associated mortalities and morbidities. This retrospective single-center study focused on intrathoracic anastomotic leakages following transthoracic esophageal resections.

### Patients and methods

The reporting of this study conforms to the STROBE statement [40].

### Study design and population

This single-center study was conducted at the Interdisciplinary Endoscopy Center of the University Hospital of Heidelberg. Data from all patients during the study period from March 2014 to September 2019 were prospectively collected and retrospectively analyzed. The study protocol conformed to the Declaration of Helsinki. It was approved by the Institutional Ethics Committee of the University of Heidelberg on 16.04.2014 (S-635/2013). A written informed consent was obtained from each patient or from his legally authorized representative.

Unlike several other retrospective studies on EVT, the inclusion criteria for this homogenous study population were only on intrathoracic anastomotic leakages after transthoracic esophageal resections and subsequent treatment with EVT. Anastomotic leakage was diagnosed either by endoscopy, computed tomography (CT) scan, detection of gastrointestinal content or methylene blue after oral application via drains or by air outlet via drains after air insufflation during EGD. Patients with persistent leakage after prior revisional surgery (n = 2) or prior placement of a fully-covered, self-expanding metal stent (SEMS, n = 4) (“Niti-S™ Esophageal Stent”, TaeWoong Medical, South Korea) were also included in this study. Exclusion criteria included etiologies for esophageal leakages other than postoperative genesis. Examples of these are spontaneous or iatrogenic causes. Initial operations other than transthoracic esophageal resections were also excluded. The patients’ demographic and clinical characteristics were retrieved. Furthermore, the leakage characteristics, surgical, and EVT procedural data were collected and analyzed.

The primary endoscopic treatment modality for anastomotic leakages in our institution was SEMS placement before the established use of EVT. In 2015, EVT was introduced into clinical routine use at our institution and we changed our endoscopic first-line treatment from SEMS placement to EVT.

### Primary and secondary endpoints

The primary endpoint of the study was a successful leakage closure rate with EVT. This was defined as endoscopically-verified resolution of the leakage and the presence of surface epithelium on the former defect. EVT failure was defined as follows: persistent leakage or fistula after termination of EVT; change of treatment strategy; need for surgical reoperation due to anastomotic leakage after EVT; and, death before confirmation of healing.

Secondary endpoints included the feasibility of the EVT procedure (application of the sponge), duration of EVT, number and frequency of sponge changes, procedure-related complications of EVT (i.e. endoscopic treatment-associated complications such as bleeding and peri-interventional associated complications such as aspiration and oxygen desaturation during sedation), duration of intensive care unit (ICU) and intermediate care unit (IMC) hospitalization, overall hospital stay length, course of inflammatory markers during EVT (i.e. white blood cell (WBC) count and levels of c-reactive protein (CRP)) and procedure-related and in-hospital mortality.

### Surgical procedure of abdomino-thoracic esophageal resection

All patients underwent elective esophageal resection with abdomino-thoracic incision, combined with immediate reconstruction, using a tubularized stomach. The blood supply was based on the right gastro-epiploic artery [41, 42]. A combination of midline laparotomy and right thoracotomy was performed for esophageal resection and two-field lymphadenectomy. For the intrathoracic anastomosis at the apex of the chest, a circular stapler was used. The stomach was divided with a linear stapler to resect the lesser curvature and the adjacent lymph nodes [43, 44]. A nasogastric tube was placed into the proximal tubularized stomach and two basal pleural drains were inserted. The postoperative patient management was standardized. From days 1 to 5, only water and tea were allowed. From day 6 onwards, the oral intake was increased in a stepwise fashion.

### Surgical procedure of esophageal diversion

Esophageal diversion was in general performed as an abdomino-cervical approach. From the abdomen transhiatal the intrathoracic anastomosis was disconnected and the thoracic esophagus was dissected bluntly or using an energy dissection device. A percutaneous feeding tube was inserted into the remaining gastric tube. The cervical esophagus was transected from the left side via an oblique cervical incision. The proximal esophagus was exteriorized via the cervical incision and an esophago-cutaneostomy was constructed at the level where the esophagus is macroscopically well perfused.

### Endoscopic vacuum therapy (EVT)

**Fig. 1:**
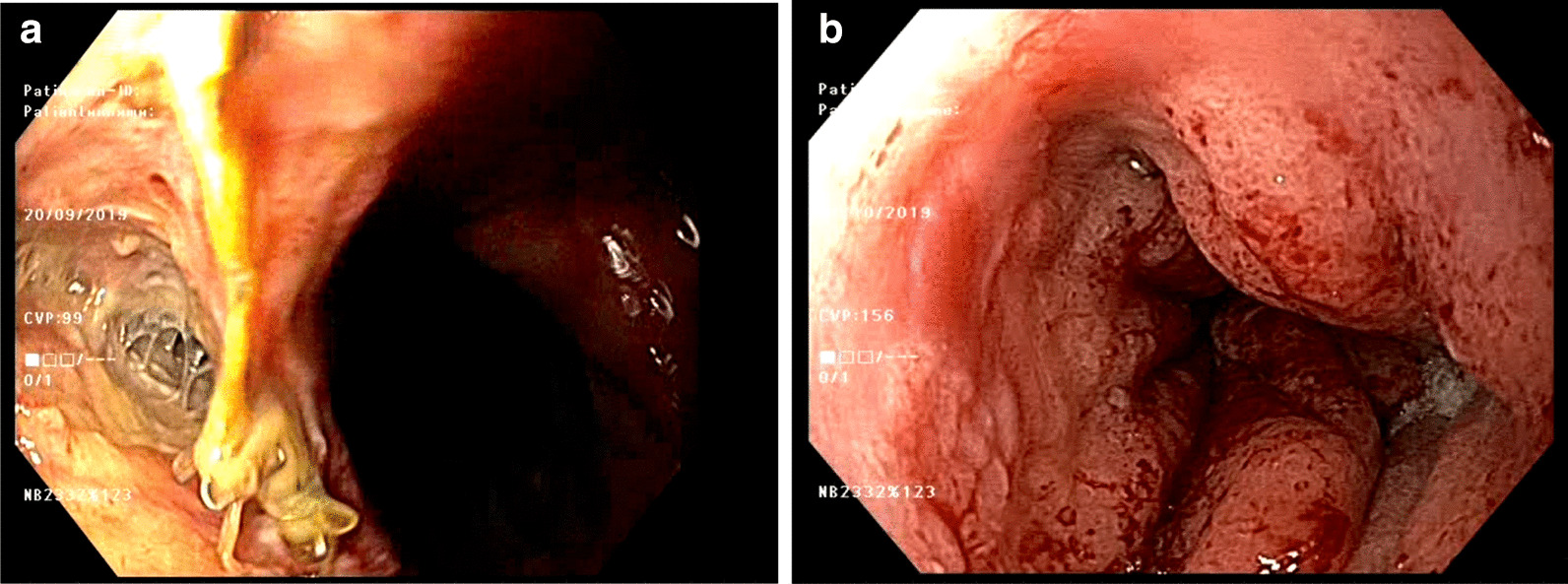
Anastomotic leakage and treatment by endoscopic vacuum therapy (EVT). **a** Anastomotic leakage before treatment. **b** Closure of the defect and recovery of the mucosal surface after EVT

Endoscopic vacuum therapy was performed as previously reported and described by Wedemeyer et al. [37, 45]. If the leakage size was large enough and the cavity was accessible to the endoscope, the sponge was inserted into the abscess cavity (intracavitary localization of the sponge). The intracavitary localization of the sponge was documented by CT scan at the beginning of EVT. It was adjusted in case there was close proximity to large vessels. With a diminishing defect size, the sponge was transferred from its initial intracavitary position into an intraluminal position. For cases with only small defects which could not be passed by the endoscope initially, the sponge was placed into the lumen of the esophagus to cover the leak (intraluminal localization of the sponge). The intracavitary-positioned sponge was changed after 3 days. The intraluminal-positioned sponge was changed after 5–7 days. These changes were done until the defect size became too small for further sponge placements and until the defect was lined with surface epithelium (Fig. 1).

The endoscopic procedures were performed either under conscious sedation or general anesthesia, depending on the medical condition of each patient. If pleural drains were extracted at the time of the leakage diagnosis and the sponge was placed intraluminally, the decision for additional external drainage was based on the CT-scan report and the clinical course of the patient.

Generally, in our institution, the further management of anastomotic leakage was based on the size and etiology of the leak, the degree of local contamination, and the severity of the associated systemic response. The decisions for further treatment (i.e. conservative, endoscopic (SEMS, EVT), or operative) were made by a group consisting of a surgeon, surgical endoscopist, and anesthesiologist. In 2014, this special team was established and tasked with decision-making regarding cases of suspected leakage in the early postoperative period after esophageal resections to optimize the clinical management of these patients [46].

### Statistical Analysis

Descriptive statistics were calculated for all parameters. The results were expressed as means ± SD (standard deviation) or as median and interquartile ranges for continuous variables. For categorical variables, counts and percentages were used. The student’s t-test was used to compare the differences between means and medians. A *p* ≤ 0.05 was considered statistically significant. The analyses were performed using the SPSS software (SPSS Inc., Chicago, IL version 1.07).

## Results

**Fig. 2:**
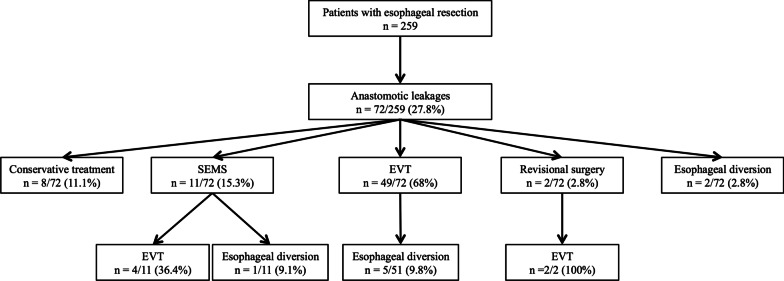
Study flow chart. Different treatment strategies of patients with anastomotic leakage during the study period. EVT: endoscopic vacuum therapy, SEMS: self-expanding metal stent

A total of 259 patients (217 men, 42 women) with a mean age of 62 years underwent elective esophageal resection through an abdomino-thoracic incision at our institution from March 2014 to September 2019 (Fig. 2). Seventy-two patients (27.8%) suffered from an anastomotic leakage. Of these 72 patients, eight (11.1%) were treated conservatively. Eleven patients (15.3%) received SEMS therapy. EVT was applied as the primary therapy for anastomotic leakage in 49 patients (68%). Two patients (2.8%) received revisional surgery while another two patients (2.8%) underwent esophageal diversion. A change of therapy was encountered in six patients, who were either initially treated by SEMS (n = 4) or had an unsuccessful revisional surgery (n = 2) and received EVT later. Six patients underwent esophageal diversion after unsuccessful EVT (n = 5) or SEMS therapy (n = 1). The overall successful treatment of all anastomotic leakages was achieved in 87.5% of patients (n = 63). The overall collective mortality rate was 3.9% (n = 10).

### Demographics of the EVT collective

Fifty-five patients (46 men, 9 women) with a median age of 63 years (range 38–82 years) were treated with EVT for intrathoracic anastomotic leakages (Table 1). Forty-nine patients (89.1%) received EVT as the first-line therapy, while six patients (10.9%) were treated with EVT after revisional surgery and persistent leakage (n = 2; 3.6%) or failure of SEMS treatment (n = 4; 7.3%). All 55 patients underwent elective Ivor-Lewis esophageal resection due to malignancies of the esophagus (SCC: n = 7, 12.7%; AEG: n = 47, 85.5%; and, GIST: n = 1, 1.8%). A total of 46 patients (83.6%) received neoadjuvant therapy: 37 patients (67.3%) underwent chemotherapy prior to surgery and 9 patients (16.4%) received combined neoadjuvant radiochemotherapy. The mean endoscopically-detected leakage size was 10 mm ± 9 mm with a median of 5 mm (range 1–30 mm). These were consecutively graded at the time of diagnosis into small defects at 0–9 mm (n = 34; 61.8%), intermediate defects at 10–20 mm (n = 14; 25.5%), and large defects > 20 mm (n = 7; 12.7%).

**Table 1:** Patient demographics

Overall patients, n	55
Age, years	
Mean ± SD	62 ± 22
Median	63
Min	38
Max	82
Gender, n (%)	
Male	46 (83.6)
Female	9 (16.4)
Indication for surgery prior to EVT, n (%)	
Malignancy	55 (100)
SCC	7 (12.7)
AEG	47 (85.5)
GIST	1 (1.8)
Neoadjuvant therapy, n (%)	
Total	46 (83.6)
CTx	37 (67.3)
RCTx	9 (16.4)
Leakage size, mm	
Mean ± SD	10 ± 9
Median	5
Min	1
Max	30
Leakage grade*, n (%)	
Small	34 (61.8)
Intermediate	14 (25.5)
Large	7 (12.7)
Procedures prior EVT, n (%)	
None	49 (89.1)
Revisional Surgery	2 (3.6)
SEMS	4 (7.3)

EVT: endoscopic vacuum therapy, GIST: gastrointestinal stromal tumor, SCC: squamous cell carcinoma, AEG: adenocarcinoma of esophago-gastric junction, CTx: chemotherapy, RCTx: radiochemotherapy, SD: standard deviation, SEMS: self-expanding metal stent

* Grading of defect size performed at initial endoscopy: small: 0–9 mm; intermediate 10–20 mm; large > 20 mm

### Feasibility of Endoscopic vacuum therapy (EVT)

EVT data are shown in Table 2. Technically, EVT could be performed on all patients included in this study. The method is easily applicable and can be learned quickly. In total, 272 polyurethane sponges were inserted in this study population. Defects were detected at a median of 7 postoperative days (range, 0–29). The median duration of EVT was 14 days (range, 3–60) with a median number of 3 EVT procedures per patient (range, 1–14). Forty-four patients (80%) received an intraluminal sponge placement at the initiation of EVT therapy. In 11 patients (20%), the sponge was placed primarily into the cavitary. Later, it was transferred into an intraluminal position during the course of the healing process upon a diminishing defect size. The median length of the intensive care unit (ICU) and intermediate care unit (IMC) stay was 23 days (range, 2–106 days). The median overall hospital stay was 39 days (range, 17–109 days).

**Table 2:** Endoscopic vacuum therapy data

First EVT, postoperative days	
Mean ± SD	7.8 ± 5
Median	7
Min	0
Max	29
Number of EVT per patient	
Mean ± SD	3.9 ± 2.6
Median	3
Min	1
Max	14
Localization of sponge at EVT initiation, n (%)	
Intracavitary	11 (20.0)
Intraluminal	44 (80.0)
Duration of EVT, days	
Mean ± SD	16.6 ± 10.3
Median	14
Min	3
Max	60
Length of ICU/IMC stay, days	
Mean ± SD	29.2 ± 20.4
Median	23
Min	2
Max	106
Overall length of hospital stay, days	
Mean ± SD	46.8 ± 20.8
Median	39
Min	17
Max	109

EVT: endoscopic vacuum therapy, SD: standard deviation, ICU: intensive care unit, IMC: intermediate care unit

### Effectiveness of EVT: successful closure rate and failure of therapy

The overall successful healing of the anastomotic leakages was achieved in 49 of 55 patients (89.1%). These patients had a reduction of the cavity size and closure of the leakage by granulation tissue (Table 3). Two patients with EVT as the second-line therapy after unsuccessful revisional surgery and four patients with a therapeutic switch to EVT after failure of the SEMS treatment due to insufficient sealing were treated successfully with EVT.

**Table 3:** Successful closure and failure of therapy rates of EVT for intrathoracic anastomotic leakages

Overall successful closure rate, n (%)	49 (89.1)
EVT as first-line, n (%)	42/49 (85.7)
Revisional surgery prior EVT, n (%)	2/2 (100)
SEMS prior EVT, n (%)	4/4 (100)
Failure of therapy, n (%)	6 (10.9)
Esophageal diversion	5 (9.1)
Death before confirmation of healing	1 (1.8)

EVT: endoscopic vacuum therapy, SEMS: self-expanding metal stent

The EVT overall failure rate of this study was 10.9% (n = 6). Five patients (9.1%) received an esophageal diversion: two of them had an necrosis of the conduit (3.6%); another 2 patients (3.6%) showed persistent elevated inflammatory markers during EVT and no further response to EVT during multiple endoscopic interventions; and, the last of the five patients (1.8%) suffered from symptomatic bleeding during EVT. For this patient, the CT-scan revealed a pseudoaneurysm of the thoracic aorta. It was successfully bridged with a thoracic endovascular aortic repair (TEVAR), followed by an esophageal diversion, and a successful restoration of bowel continuity. The sixth patient died during EVT. This patient suffered from an unclear cardiac arrest. The reanimation was also unsuccessful. The relatives denied autopsy; hence, the underlying cause of death remains unclear.

### Inflammatory markers during EVT

**Fig. 3:**
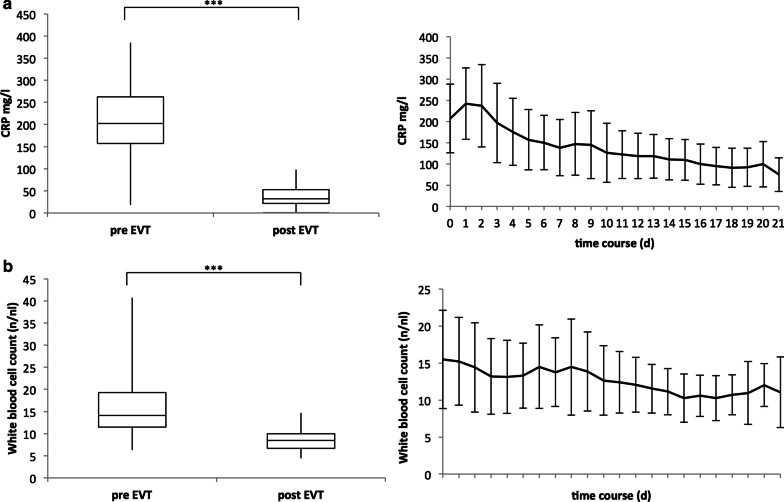
Inflammatory markers during EVT. **a** CRP levels before the beginning of EVT decrease significantly compared to CRP levels one day after the termination of EVT. **b** White blood cell count (WBC) before EVT shows a significant reduction compared to values obtained one day after the termination of EVT. Pre-EVT: values before EVT. Post-EVT: values one day after termination of EVT. *** indicates a *p* value ≤ 0.001

White blood cell count (WBC) and c-reactive protein (CRP) levels are shown in Fig. 3. CRP values (Fig. 3A) and WBC count (Fig. 3B) were measured at the beginning of EVT, daily during EVT, and during the first day following the termination of EVT. All patients showed signs of a systemic inflammatory response at the beginning of EVT with elevated CRP levels frequently accompanied by leukocytosis. The median CRP value at the beginning of EVT was 202.5 mg/l (range, 18–385 mg/l). The median WBC count at the beginning of EVT was also elevated at 14 /nl (range, 6–41 /nl). Both inflammatory markers decreased during EVT treatment. The median CRP value was 30.5 mg/l on the first day following the termination of EVT (range, 2–98 mg/l) while the median WBC count was 8.5 /nl (range, 4–14.7 /nl). Both inflammatory markers showed a significant reduction after the termination of EVT compared to values at the beginning of EVT (CRP: *p* ≤ 0.001; WBC: *p* ≤ 0.001).

### Safety of EVT: Procedure-related and in-hospital morbidity and mortality

As shown in Table 4, the overall EVT-associated complication rate in our study was 5.4% (n = 3): One complication (1.8%) during EVT was the minor bleeding of the above-mentioned patient which led to the diagnosis of an aortoesophageal fistula successfully treated by TEVAR and esophageal diversion. This patient recovered fully thereafter and achieved restoration of the bowel continuity by a colon pull-up eight months later. Minor procedural complications occurred in 2 other patients (3.6%) during EGD with conscious sedation (oxygen desaturation in one patient and aspiration in the other). Both patients recovered fully with intermittent assisted ventilation via respiratory mask after the termination of endoscopy. They underwent the EVT procedure under general anesthesia thereafter without any further complications.

**Table 4:** Overall complication and in-hospital mortality rates

Overall complication rate, n (%)	3 (5.4)
Bleeding	1 (1.8)
Oxygen desaturation	1 (1.8)
Aspiration	1 (1.8)
In-hospital mortality, n (%)	4 (7.2)
Cardiac arrest	1 (1.8)
Multi-organ failure	1 (1.8)
Urosepsis	1 (1.8)
Severe ARDS	1 (1.8)

ARDS: acute respiratory distress syndrome

The in-hospital mortality rate was 7.2% (n = 4). In contrast to the above-mentioned patient who suffered a cardiac arrest under ongoing EVT, three other patients died after successful EVT and endoscopically-assured leakage closure. The causes of deaths in these patients were septic shock with multi-organ failure (MOF), urosepsis, and severe acute respiratory distress syndrome (ARDS), respectively.

## Discussion

This study indicates the feasibility, effectiveness, and safety of EVT for intrathoracic anastomotic leakages after esophageal resections. For many years, the placement of self-expanding stents had served as the first-line therapy for anastomotic esophageal leakages [26–29]. Several studies had indicated the effectiveness and safety of stent therapy exclusively on the basis of non-randomized evidence [26, 27, 29–31]. However, the reported mortality rates of stent therapy had varied widely from 0 to 83% [13, 32–35]. The reported failure rate of stent therapy had been about 15–30%. These rates included mortalities despite stent therapy and failures of anastomotic healing [27, 29–31].

Recently, there has been growing evidence on the effectiveness of EVT as an initial therapy for anastomotic leakages after esophageal resections. Although in contrast to stent placement, EVT requires multiple endoscopic procedures, there are several advantages of EVT over stent therapy: the vacuum system provides optimal drainage of the wound and an effective sepsis control in case of mediastinitis. Perfusion is also promoted. These culminate in granulation formation and healing of the defect [36]. The wound cavity can be visualized regularly and deterioration may be detected early.

Nevertheless, data regarding EVT for intrathoracic anastomotic leakages are still rare. Previously published studies were mostly case reports. These considered small patient cohorts with only 1–39 patients [12, 39, 47]. The reported success rates of EVT for anastomotic leakages are high (66–100%) [12, 13, 36, 39, 47–58]. Accordingly, complication rates are low (0–14.8%) [12, 39, 47, 51, 52]. These data are consistent with the results of our current study, which indicates a success rate of 89.1% and an overall procedure-related complication rate of only 5.4%.

The observed successful closure rates and effective sepsis control of EVT were accompanied by a rapid decrease in inflammation markers (CRP and white blood cell count). In accordance with the study results of Laukoetter et al. [12], we showed a significant decrease in both inflammatory markers compared to initial values after the termination of EVT.

Several studies show higher success rates of endoscopic vacuum therapy compared to stent placement for anastomotic leakages. Berlth et al. included only patients after upper GI surgery for malignancies and compared 77 SEMS-treated patients with 34 EVT-treated patients [47]. The success rate of EVT was more favorable at 85.7% compared to SEMS treatment (72.4%). However, this result was not statistically significant. This study included the highest number of patients to date. Although only patients with anastomotic leakages after oncological gastroesophageal surgery were analyzed, it included not only patients with intrathoracic anastomosis, but also patients with cervical and abdominal leakages.

A significantly higher closure rate of EVT compared to SEMS therapy for treatment of intrathoracic leakages was found by Brangewitz et al. (84.4% vs. 53.8%, respectively) [51]. The authors compared 39 stent-treated patients with 32 EVT-treated patients. They also detected a higher stricture rate after SEMS treatment. However, the patients’ cohorts of this study were heterogeneous since patients who underwent esophageal resection, gastric fundoplication, iatrogenic perforation, and those who developed Boerhaave syndrome were included in the analyses.

Three further studies with only small cohorts compared the outcomes of SEMS and EVT for anastomotic leakages following esophageal resection or gastrectomy: Schniewind et al. showed significantly lower mortality rates for EVT (n = 17) compared to those for SEMS (n = 6) in systemically ill patients (12% vs. 83%, *p* = 0.0014) [13]. The study results of Mennigen et al. indicated a higher success rate of anastomotic healing for 15 EVT-treated patients compared to 30 SEMS-treated patients (93.3% vs. 63.3%) [36]. Hwang et al. reported higher success rates of EVT (100%, n = 7) compared to SEMS (63.6%, n = 11) [59]. A lower complication rate for EVT was also noted (0% vs. 54.5%).

Recently, Rausa et al. performed a meta-analysis of four studies and found significantly higher closure rates, shorter treatment durations, and lower complication and mortality rates for EVT compared to those for SEMS for esophageal leakages of different etiologies [60].

Our current study only included patients with intrathoracic anastomotic leakages after esophageal resections. We focused only on abdomino-thoracic incisions. In our study population, we showed a successful EVT closure rate of 89.1%. Compared to our own homogenous historic patient cohort (n = 32) (article in preparation) about intrathoracic anastomotic leakages following abdomino-thoracic esophageal resection and treatment with SEMS placement, a success rate of 62.5% was detectable. This was notably lower than the success rate of EVT in our study (89.1%). Overall, the in-hospital mortality rate of patients with EVT therapy in our study was 7.2% while the mortality rate in our historic SEMS-treated patient cohort was clearly higher at 15.7%. These data are consistent with the above-mentioned studies. They indicate a favorable effect of EVT compared to stent placement in terms of successful closure, complications, and mortality rates for intrathoracic anastomotic leakages [36, 51, 59].

Although the data from our SEMS group are not novel, these definitely compare two homogenous patient populations with only intrathoracic anastomotic leakages after abdomino-thoracic esophageal resections. These procedures were performed at our institution according to the same standardized procedures. Although the success rate of 62.5% in our stent cohort was at the lower end of the reported success rates of other groups evaluating only SEMS therapy for esophageal leakages [26, 30, 31], it is in congruence with studies comparing EVT and SEMS placement [36, 51, 59]. All comparative studies have the same limitations. A historical SEMS-treated patient cohort served as the comparison group. This may have led to a disruption of stent therapy at an earlier stage as soon as EVT was available. Without EVT serving as the alternative therapeutic option, the SEMS treatment would likely have been continued to avoid esophageal diversion.

With regards to the prospectively collected data, but retrospective analysis our cohort study has certain limitations. These include the non-randomized design and limited number of patients included. On the other hand, no prospective randomized or comparative trials for this topic have been published. Our study presents the largest single-center cohort of a homogenous population of EVT-treated patients suffering intrathoracic anastomotic leakage after abdomino-thoracic esophageal resection. Apart from this, considering that the above-mentioned studies showed an apparent superiority of EVT compared to stent therapy, one can doubt how ethical a randomized trial in this context might be.

## Conclusion

Endoscopic vacuum therapy is a feasible, effective, and safe method of endoscopic treatment for intrathoracic anastomotic leakages following abdomino-thoracic esophageal resections. This strategy leads to high rates of anastomotic healing and has the potential to reduce the overall mortality.

## Data Availability

The datasets generated during and analysed during the current study are not publicly available due to compromise of individual privacy but are available from the corresponding author on reasonable request.

## Abbreviations

AEG: Adenocarcinoma of esophago-gastric junction
AL: Anastomotic leakage
ARDS: Acute respiratory distress syndrome
CRP: C-reactive protein
CT: Computed tomography
EVT: Endoscopic vacuum therapy
GI: Gastrointestinal
MOF: Multiple organ failure
OTSC: Over-the-scope clip
SCC: Squamous cell carcinoma
SEMS: Self-expanding metal stent
TEVAR: Thoracic endovascular aortic repair
WBC: White blood cell count
